# 

**Published:** 1897-07

**Authors:** 


### Southern Medical Record.

A Monthly Journal of Medicine and Surgery.

VOL. XXVII. ATLANTA, GA., JULY, 1897. No. 7

BERNARD WOLFF, M. D., EDITOR.
ASSOCIATE EDITORS:
A. W. GRIGGS, M. D. WILLIS F. WESTMORELAND, M. D.
J. McFADDEN GASTON, M. D.
E. Y. CLARKE, GENERAL MANAGER. B. M. ZETTLER, BUSINESS MANAGER.

Entered at the Post-office at Atlanta, Ga., as Second-class Matter.

The Foote & Davies Co., Atlanta, Ga.

